# Promoting health information system in guiding decisions for improving performance: an intervention study at the Research Institute of Ophthalmology, Giza, Egypt

**DOI:** 10.3389/fdgth.2024.1288776

**Published:** 2024-09-18

**Authors:** Yara H Abdelgawad, Madiha Said Abd El Razik, Doa’a A Saleh, Manal H Abuelela, Marwa Rashad Salem

**Affiliations:** ^1^ Department of Public Health and Community Medicine, Research Institute of Ophthalmology, Giza, Egypt; ^2^ Department of Public Health and Community Medicine, Faculty of Medicine, Cairo University, Cairo, Egypt

**Keywords:** operations research, KPI, MIS, performance index, decision matrix

## Abstract

**Objectives:**

This study aims to design and test a platform of key performance indicators (KPIs) and indices emphasizing achievements and improvement and helping decision-making.

**Methods:**

An operations research study was designed to analyze data from the Hospital Management Information System (HMIS) from July 2017 to June 2018 at the Research Institute of Ophthalmology (RIO), Giza, Egypt. The HMIS data were submitted to reform covering parameters in service delivery and corresponding indicators and indices. Data were grouped into four themes: human resources and outpatient, inpatient, and surgical operations. A total of 14 performance indicators were deployed to four specific indices and total performance indices and applied to six teams of ophthalmologists at RIO. The decision matrices were deliberated to demonstrate achievements and provide recommendations for subsequent improvements.

**Results:**

Throughout 1 year, six teams of ophthalmologists (*n* = 222) at RIO provided the following services: outpatient (*n* = 116,043), inpatient (*n* = 8,081), and surgical operations (*n* = 9,174). Teams 2, 1, and 6 were the top teams in the total performance index. Team 4 had plunges in the outpatient index, and Team 5 faced limitations in the inpatient index.

**Conclusion:**

The study provided a model for upgrading the performance of the management information system (MIS) in health organizations. The KPIs and indices were used not only for documenting successful models of efficient service delivery but also as examples of limitations for further support and interventions.

## 1 Introduction

A health information system (HIS) is a system designed to manage healthcare data. This includes systems that collect, store, manage, and transmit the electronic medical records (EMR) of patients, hospital operational management, or healthcare policy decisions. HIS also includes those systems that handle data related to the activities of healthcare providers and health organizations (1). As an integrated effort, those systems may be produced to improve patient outcomes, inform research, and influence policy-making and decision-making (2).

Hospitals, as highlighted in the World Health Report, are significant healthcare providers and one of the factors determining reasonable healthcare distribution and the promotion of the justice index in the healthcare system by serving as a vital foundation for clinical research and facilitating healthcare coordination and integration (1). Furthermore, improved hospital performance helps health systems achieve their intermediate and final goals at all levels (3).

A hospital management information system (HMIS) is pivotal for the management of patient care services and related administrative functions to handle all domains of the operation of a hospital. It has the potential to improve the efficiency (proper utilization of resources to achieve maximum output) of the overall system through automation and generation of necessary reports for managing operations, performance, quality, planning, and decision-making (4). In health organizations involved in patient tertiary care and research, health reports about patients are crucial for epidemiological studies as risk assessment through cohort studies and case–control studies. Those organizations could also have supportive HMIS for the conduction of clinical trials, related medications, and surgical operations (5). For those reasons, HMIS should be properly designed to match the mission, goals, strategic objectives, and the operation of functions of an organization (6). During the planning stage for finalizing HMIS implementation, all the stakeholders of the hospital should be involved to confirm that all necessary features and functions are available in the system to manage their workflow (7).

The HMIS is an indispensable system for generating monitoring and evaluation reports and providing feedback for decision-making that is used as a strategy for changing the clinical practice and behaviors of healthcare personnel (2). Performance assessment and feedback are intended to enhance professional performance and thereby improve the quality of health care and patient safety (8).

For successful HMIS, training in the appropriate module of the HMIS users is one of the important processes in the HMIS execution (9). There is a need for standardization of the workflow and assurance that the software supports the workflow (10). Data validation and quality are essential for HMIS (11). Successful HMIS for specific organizations could be scaled up to be used in similar organizations at national and international levels (12).

Despite the importance of HMIS, many of the hospitals do not use the HMIS beyond automation of services and daily reporting while some of the users use it extensively for continuous improvement (13). The periodical reports and communicating information in the form of indicators and indices as well as getting feedback of information from teams of health service providers could overcome barriers to improve performance (14).

The Research Institute of Ophthalmology (RIO) is a leading research center of ophthalmology in Egypt. It has a strong HMIS with extensive and comprehensive data since 2016 to build a database for research, enhance evidence-based decision-making, and retrieve the data reflecting the performance of the hospital easily. This is used in addition to its medical records. However, the HMIS had many limitations related to the design of the system and disorganized data that align with the mission and objectives of RIO. Additionally, the operating teams faced challenges due to the inability to provide periodic reports that demarcate functions according to key performance indicators (KPIs). There is poor compliance from some physicians in data entry. In RIO HMIS, there are no KPIs to measure achievements and shortcomings in service delivery. Retrieval of the stored data could not provide indicators as the data are physician-oriented/concerned. There are no patient-oriented or team-oriented folders. There are no models for reports to communicate information with service providers for decision-making and upgrading performance. Therefore, our study was conducted to develop strategies to promote the role of HMIS in data utilization and to establish communication channels for exchanging information with service providers. These initiatives are crucial for guiding decisions aimed at improving performance. Having well-defined KPIs and indices allows the use of standardized methods for monitoring and evaluating performance across the RIO ophthalmologist teams. Thus, this enables healthcare providers to become aware of their performance relative to each other, points of strength, and sub-optimal performance to adopt strategies for improving performance.

Previous studies focused on developing hospital KPIs (15), utilizing the balanced scorecard (16) and the analytic network process models (17). Other studies investigated the effectiveness of the HIS in improving performance from the perspective of clinical staff (18). Most studies explored the factors affecting HIS implementation (19, 20).

A previous study conducted at RIO revealed that the HIS implementation plan provided the necessary information for each patient (21), while our current study aims to improve the performance of RIO by upgrading the MIS.

Study hypothesis: The presentation and discussion of the performance matrix through organizing RIO HMIS data and development of KPIs for RIO throughout 1 year then the alignment of each group of indicators into specific parameters related to sets of services. This is followed by estimating the performance index for each parameter and the total performance index, which will inform policymakers at different levels about the achievements and suggested interventions for improving performance.

The goal of the current study is to improve the performance of health organizations by upgrading the MIS.

The objectives are to construct a set of KPIs from HMIS data in RIO for 1 year, create matrices of indicators to overview the performance parameters (outpatient, inpatient, and surgical operations), and identify the factors contributing to the success and /or the sub-optimal performance of each of the six teams.

## 2 Methods

### 2.1 Study design and setting

The study was an operations research intervention posttest study conducted at RIO in Giza, Egypt, focusing on RIO HMIS computerized data organized for four quarters from July 2017 to June 2018. The institute is committed to providing healthcare services to patients from all over Egypt and supporting research in ophthalmology at national and international levels. It also provides a tertiary level of healthcare, is affiliated with the High Council of Universities, and has distinguished staff members of professional ophthalmologists. It is characterized by providing 1-day surgery and ultrashort hospital stay services. The institute offers many outstanding research and therapeutic services at reduced economic prices and free of charge for those who are unable to afford them. Covering various disciplines of eye medicine and surgery, the institute offers general clinics, diagnostic clinics, specialized clinics, surgical operations, and internal departments and laboratories.

At RIO, the staff is comprised of 450 administrative employees and 320 physicians and researchers.

The ophthalmology outpatient clinics include eight general clinics and ten specialized clinics: two surgical retina clinics, one medical retina clinic, two glaucoma clinics, and one clinic for each of the following sub-specialties: oculoplasty, cornea, pediatric ophthalmology, refractive, and cataract specialties. The radiology examination clinics offer an array of services such as laser and US biometry, OCT, VEP, ERG, and Pentacam.

Six teams of healthcare providers manage the ophthalmology outpatient clinics across six working days with an average of 34 physicians in each team. Before the year 2000, ophthalmologists were divided among five working days with the sixth day considered rotatory. Subsequently, the arrangement shifted to comprise six teams. Each team conducts nine clinical activities distributed throughout the week, with an exchange of each function across teams and throughout the week. The covered sub-specialties include general ophthalmology clinics, all the ophthalmology sub-specialties, radiological examination clinics, and surgical operations.

### 2.2 Sampling technique and sample size

All available data for the period spanning four quarters from July 2017 to June 2018 were selected. The data include information from all patients and service providers, i.e., ophthalmologists. The ophthalmologists working at RIO are allocated into six teams.

### 2.3 Type of data and data collection

All HMIS data were quantitative and computerized data in a specific program. The data were recorded for each of the six teams of ophthalmologists. The researcher identified all the variables in the HMIS and regrouped the data into a specialized format, which was transferred (imported) to an Excel program. The data were submitted for revising and quality check according to the different files of the HMIS.

### 2.4 Data analysis plan

The data were submitted for transformation into different indicators. Additionally, 14 indicators were used to develop performance indices. The indicators were categorized into horizontal and vertical types. The horizontal indicators were grounded on linking the performance of each of the six teams to the total output of RIO. In contrast, the vertical indicators were based on presenting the performance of each team separately. These indicators were further regrouped into four parameters of performance: human resources (HR), outpatients, inpatients, and surgical operations, which are described in Supplementary Data Sheet S2. Furthermore, four indices were developed for the four parameters and the total performance index (Table 1). The decision matrices were developed to inform policy makers at the central level of RIO and each of the six teams of ophthalmologists (Matrix 1) and (Supplementary Matrices S1–S5) which are described in Supplementary Data Sheet S1.

**Table 1:** Parameters of performance, indicators, and indices.

Parameters of performance	Indicators	Indices
Human resources parameter Manpower (ophthalmologists): three indicators	Percent of the total team members to the total RIO staff members	Human resource index
	Percent of professors and assistant professors within the team to the total professors and assistant professors in RIO	
	Percent of trainees and fellowship scholars within the team to the total trainees and fellowship scholars in RIO	
Outpatient services parameter: four indicators	Percent contribution of each team of ophthalmologists to the total outpatient cases throughout 1 year	Outpatient services index
	Percent contribution of each team of ophthalmologists to the total outpatient cases aged less than 25 years throughout 1 year	
	Percent contribution of each team of ophthalmologists to the total outpatient cases aged 55 years and more throughout 1 year	
	Percent contribution of each team of ophthalmologists to the total outpatient cases who attended consultation services throughout 1 year	
Inpatient services parameter: four indicators	Percent contribution of each team of ophthalmologists to total inpatient cases throughout 1 year	Inpatient services index
	Percent contribution of each team of ophthalmologists to the total inpatient cases defined as new cases (no previous admission to RIO) throughout 1 year	
	Percent of staff members in each team who recorded diagnosis in the inpatient files	
	Percent of surgical operations conducted by each team to the total inpatients (coverage by surgical operations) in RIO throughout 1 year	
Technical (surgical operations) parameter: three indicators	Percent of surgical operations (14 categories) conducted by each team to the total surgical operations conducted in RIO throughout 1 year	Surgical operations index
	Percent of surgical operations defined as “major surgery” conducted by each team to the total major surgical operations conducted in RIO throughout 1 year	
	Percent of surgical operations defined as “skilled surgery” conducted by each team to the total skilled surgical operations conducted in RIO throughout 1 year	
	Percent of surgical operations (14 categories) conducted by each team to the total surgical operations conducted in RIO throughout 1 year	

**Matrix 1:**
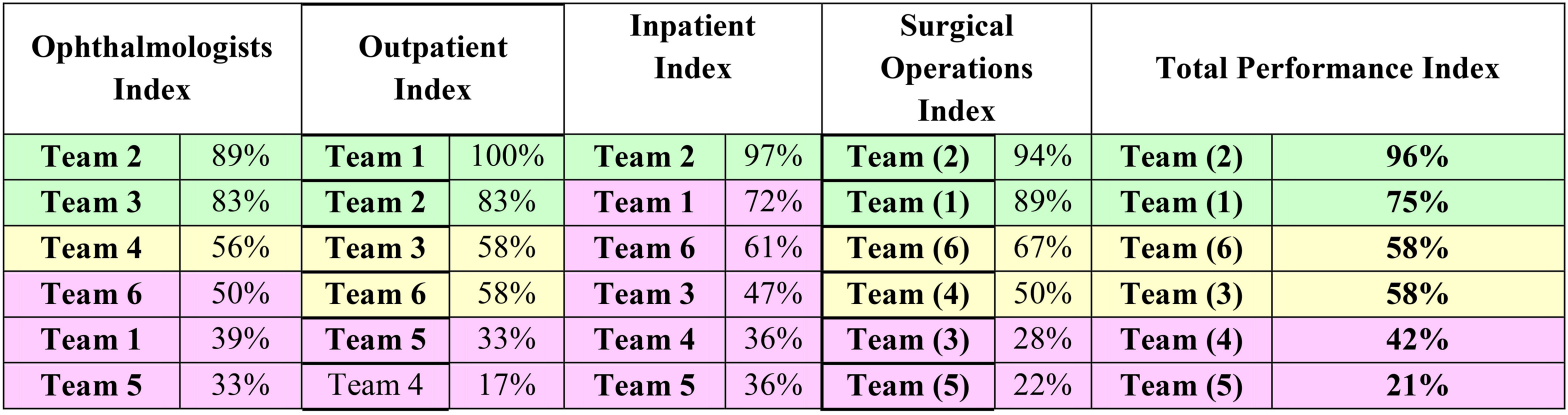
Rank ordering of each team of ophthalmologists according to indices of performance and the total performance index.

### 2.5 Performance Index

An index usually includes a base value and indicators that represent the key element to which other values will be compared. In the presented study, the developed indices had many objectives. The related indicators were grouped into four parameters to provide an index for each parameter. Then, these four parameter indices are used to provide one parameter to present one index as the total performance index. The methods of index development were dependent on recognizing the mathematical unfeasibility of adding values of indicators together, as each value is concerned with specific variables and outcomes. Therefore, the study used a new approach to have a common measure for all indicators to allow adding the new form of the indicators to facilitate adding them together.

### 2.6 Development of indices

In most health organizations, there are no set standards and/or targets to be achieved as a total performance of the organization or standard per department or team. Such situations raise difficulty in assessing the performance of the organization and its departments. The study tried to solve this issue by considering resources and workload consistently distributed across six teams. It was found that the percent contribution of each team to the total RIO services is 16.7%. Thus, a contribution of >16.7% is considered encouraging performance, and a contribution of <16.7% is considered unsatisfactory. However, other vertical indicators have to be considered in the assessment, i.e., the percentage of team members recording diagnoses in the inpatient files. The use of ranking methods was considered the reasonable method to develop indices as it allows adding different indicators together.

### 2.7 Centile method, decision matrices and total performance index

A decision matrix ranks and evaluates a set of indicators. This is done by developing indicators for each team and then analyzing each indicator against other indicators in the list and across teams. In case of the need for a single choice from several options, the decision matrix could be derived from multiple criteria and indicators (22). The current study demonstrated that the decision matrix is used in the case of having a list of indicators that must be narrowed to one choice for improvement recommendation (23).

The ranking method has to be designed for each organization as it depends on the number of departments and/or teams. For example, the current study included six teams. To develop a matrix of selected indicators of performance, the teams have to be ranked according to the value of the indicator from the highest (best situation) to the lowest (unfavorable situation). For example, the matrix of inpatient parameters included four indicators with teams sorted from the best to the least favorable situation. To develop the index for a specific parameter, the top team with the best situation was given a score of 6, and the worst team with an unfavorable situation was given a score of 1. Therefore, in the example of the inpatient parameter, each team will have four ranking scores. The total/sum rank scores for each team for specific indicators included in the parameter have to be presented as percent from the maximum score, i.e., 6 × number of indicators. The teams were rearranged according to the percentage of ranking scores from the highest to lowest and the results were input in the matrix. Categorization of matrix information was achieved by distributing the teams across indicators and indices into three categories/situations: best (green color), intermediate (yellow color), and unfavorable (red color) and by using the centile method (24, 25).

In the current study, the centile method was used as follows: the six teams were ranked from 1 to 6. The best situation level for the team, i.e., which had the highest percent contribution in any of the indicators, was provided a score of 6 and so on, according to the rank ordering of teams for each indicator. The total (sum of scores of indicators) for each team is then divided by the maximum score (6×number of indicators) to get the percent score for each team for a group of indicators. The percent scores for the teams were reallocated into three levels using centiles to have top teams, unfavorable situation teams, and intermediate-level teams. The index has no meaning in its value, but it is used as a method to add different types of indicators together and to rank each team in the special management index, e.g., HR and outpatient.

The total performance index is a composite index that includes four indices: HR, outpatient services, inpatient services, and surgical operations.

## 3 Results

Throughout 1 year, the six teams of ophthalmologists (*n* = 222) at RIO provided services as follows: outpatient (*n* = 116,043), inpatient (*n* = 8,081), and surgical operations (*n* = 9,174).

### 3.1 Manpower resources in RIO

Table 2 illustrates that the total number of physicians in RIO was 222 ophthalmologists distributed among six teams. More than a quarter of the ophthalmologists were professors and assistant professors (19% and 8%, respectively). The specialists with a master's degree in ophthalmology accounted for 18% of all staff members, while trainees and fellowship scholars accounted for 16% and 10%, respectively. The distribution of staff members by grades varied across the ophthalmologist teams. For Team 5, the professors accounted for 12% and assistant professors accounted for 15% of the team's staff members. Team 1 had 35% of its staff members graded as researchers' assistants. Team 4 had 28% of its staff members graded as researchers. The percent distribution of ophthalmologists in RIO across six teams is shown in Supplementary Figure S1, Supplementary Data Sheet 1. Out of the 222 ophthalmologists, Team 2 had the highest contribution with 20%) of the staff members, whereas Teams 1, 5, and 6 each accounted for 15% of the staff members of the total number of ophthalmologists in RIO. With reference to the total professors and assistant professors (*n* = 59) in RIO, Supplementary Figure S2, Supplementary Data Sheet 1 indicates that 20% in this category of staff members were affiliated with Team 3, and 14% were affiliated with Team 4 (Table 2).

**Table 2:** Percent distribution of each of the six teams of ophthalmologists according to staff members’ professional grades, from July 2017 to June 2018 at the Research Institute of Ophthalmology (RIO).

Staff Members Grades	Team 1	Team 2	Team 3	Team 4	Team 5	Team 6	Total	Percent
Professor(current)	9%	2%	12%	14%	6%	9%	19	9%
Professor (emirate)	12%	18%	10%	3%	6%	12%	23	10%
Assistant professor	6%	2%	7%	6%	15%	12%	17	8%
Researcher	9%	18%	20%	11%	9%	3%	27	12%
Researcher's assistant	35%	14%	12%	8%	12%	21%	37	17%
Specialist	15%	16%	15%	25%	24%	15%	40	18%
Trainees	9%	16%	7%	28%	15%	24%	36	16%
Fellowship scholars	6%	14%	17%	6%	12%	6%	23	10%
Total	34	44	41	36	33	34	222	100%

**Figure 1:**
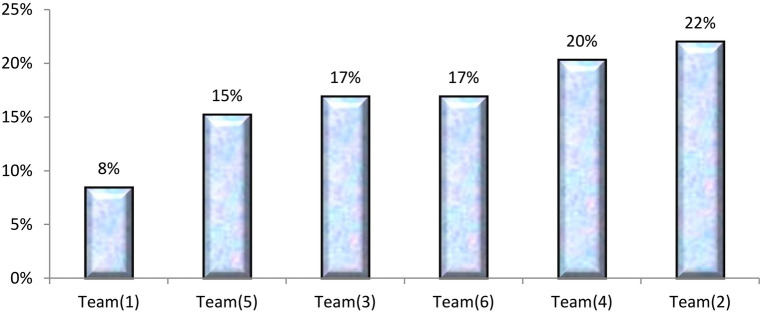
Rank order of six teams of ophthalmologists according to the proportion of the total staff members categorized as trainees and fellowship scholars (*n* = 59) at the Research Institute of Ophthalmology (RIO), July 2017–June 2018.

The staff members were categorized as trainees and fellowship scholars (*n* = 59) distributed across the six teams of ophthalmologists as demonstrated in Figure 1. Out of the total staff members in this category, 22% were affiliated with Team 2, and 8% were affiliated with Team 1 (Figure 1).

### 3.2 Outpatient services in RIO

Throughout 1 year (July 2017–June 2018), the total number of outpatients in RIO clinics was 116,043 patients. The outpatients were distributed across the four quarters of the reference year as 27%, 26%, 26%, and 21%. The configuration of the percent contribution of the six teams of ophthalmologists to the total outpatients for each team tended to be constant across the four quarters of the reference year. The percent contribution of the six teams of ophthalmologists to the total outpatient services throughout the reference year is illustrated in Table 3. The highest contribution in outpatient services was reported for Teams 1 and 2 (20.7%), and the lowest contribution to total outpatient services was for Team 4 (13%). The percent distribution of outpatients by age category (age in years) for the total outpatients and for each team is also illustrated. It is obvious that 34% of patients were in the age group 55 + years and 29% were in the age group < 25 years. At the team levels, the age group 55+ years accounted for 31% and 36% for Teams 6 and 5, respectively. Outpatients in the age group <25 years accounted for 27% and 32% for Teams 1 and 5, respectively. Ninety percent of the patients received consultation services compared to only 10% who received follow-up services. The situation at the teams' level varied between 87% and 92% for the proportion of patients who received consultation services and from 8% to 13% for follow-up services (Table 3).

**Table 3:** Percent contribution of the six teams of ophthalmologists in total outpatient services throughout 1 year (July 2017–June 2018) and percent distribution of outpatients by age and type of outpatient services for each of the teams for the same year at the Research Institute of Ophthalmology (RIO) (total outpatients = 116,043).

Outpatient services	Team 1	Team 2	Team 3	Team 4	Team 5	Team 6	
Age	Team 1	Team 2	Team 3	Team 4	Team 5	Team 6	Total
<25	27%	28%	28%	31%	32%	31%	28,513 (29%)
55+	35%	35%	33%	33%	36%	31%	32,800 (34%)
Total	19,278	19,155	14,612	13,675	13,791	16,699	97,210
Type of services	Team 1	Team 2	Team 3	Team 4	Team 5	Team 6	Total
Consultation	88%	87%	87%	92%	92%	92%	1,04,261 (90%)
Follow-up	12%	13%	13%	8%	8%	8%	11,782 (10%)
Total year 2017–2018	24,044 (20.7%)	24,020 (20.7%)	18,025 (15.5%)	15,058 (13%)	16,115 (14%)	18,781 (16.1%)	1,16,043 (100%)

Missed data of age = 16%.

### 3.3 Inpatient services in RIO

**Figure 2:**
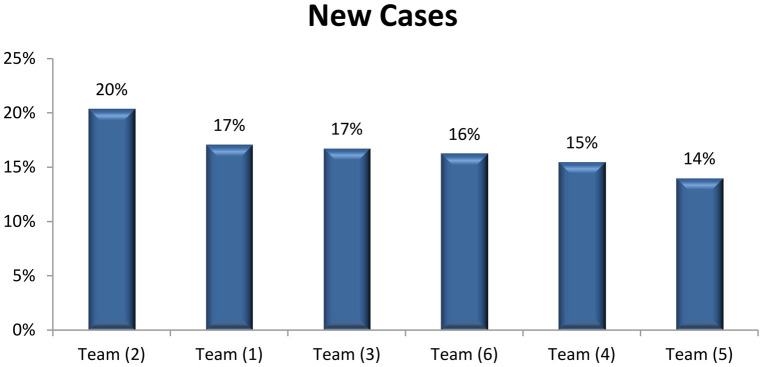
Rank order of each team of ophthalmologists according to the percent contribution of the total inpatients defined as new cases at the time of admission throughout 1 year (July 2017–June 2018) at the Research Institute of Ophthalmology (RIO).

Patients admitted for inpatient services were categorized as new cases, readmission within 72 h of discharge from RIO, and readmission after 72 h of discharge from RIO. Table 4 illustrates the percent contribution of each team of ophthalmologists in the total inpatients by the year's quarters. The indicator set as the percent contribution of the team in total admissions per quarter is a monitoring of performance indicator. In Quarter 1, each of Teams 1 and 3 contributed by 18%. In Quarter 2, each of Teams 1, 2, and 4 contributed by 19%. In Quarters 3 and 4, Team 2 contributed by 22% and 27%, respectively, and showed that the majority of inpatients were new cases (85.7%) with a range of 84% to 90.1% among all teams. Team 2 had the highest contribution (21%) in the total inpatient services throughout 1 year followed by Teams 1 and 3 (17%). About 15% (14.3%) of patients were readmissions after 72 h from discharge from RIO with a range of (9.9% to 16%) among all teams. Figure 2 demarks the rank order of the six teams of ophthalmologists in the total inpatients defined as new cases (Figure 2). Out of the total new inpatient cases, 20% and 14% were admitted by Teams 2 and 5, respectively (Table 4).

**Table 4:** Percent contribution of each team of ophthalmologists in total inpatient services throughout 1 year (July 2017–June 2018) and inpatients by type of admission across six teams of ophthalmologists throughout the same year at the Research Institute of Ophthalmology (RIO).

Admissions	Team 1	Team 2	Team 3	Team 4	Team 5	Team 6	Total
New cases	86.2%	84.0%	84.7%	84.8%	84.9%	90.1%	85.7%
Readmission < 72 h	0.7%	0.8%	0.8%	0.8%	0.7%	1.8%	0.9%
Readmission > 72 h	13.8%	16.0%	15.3%	15.2%	15.1%	9.9%	14.3%
Quarters of 2017–2018	Team 1	Team 2	Team 3	Team 4	Team 5	Team 6	Total
Q1	18%	17%	18%	17%	15%	15%	2,039
Q2	19%	19%	16%	19%	14%	13%	2,450
Q3	17%	22%	14%	15%	17%	15%	2,136
Q4	12%	27%	22%	10%	10%	20%	1,456
Total inpatients Year 2017–2018	17%	21%	17%	16%	14%	15%	100%
	1,372	1,680	1,370	1,266	1,143	1,250	8,081

Q, quarter.

The total number of hospital beds was 70.

**Figure 3:**
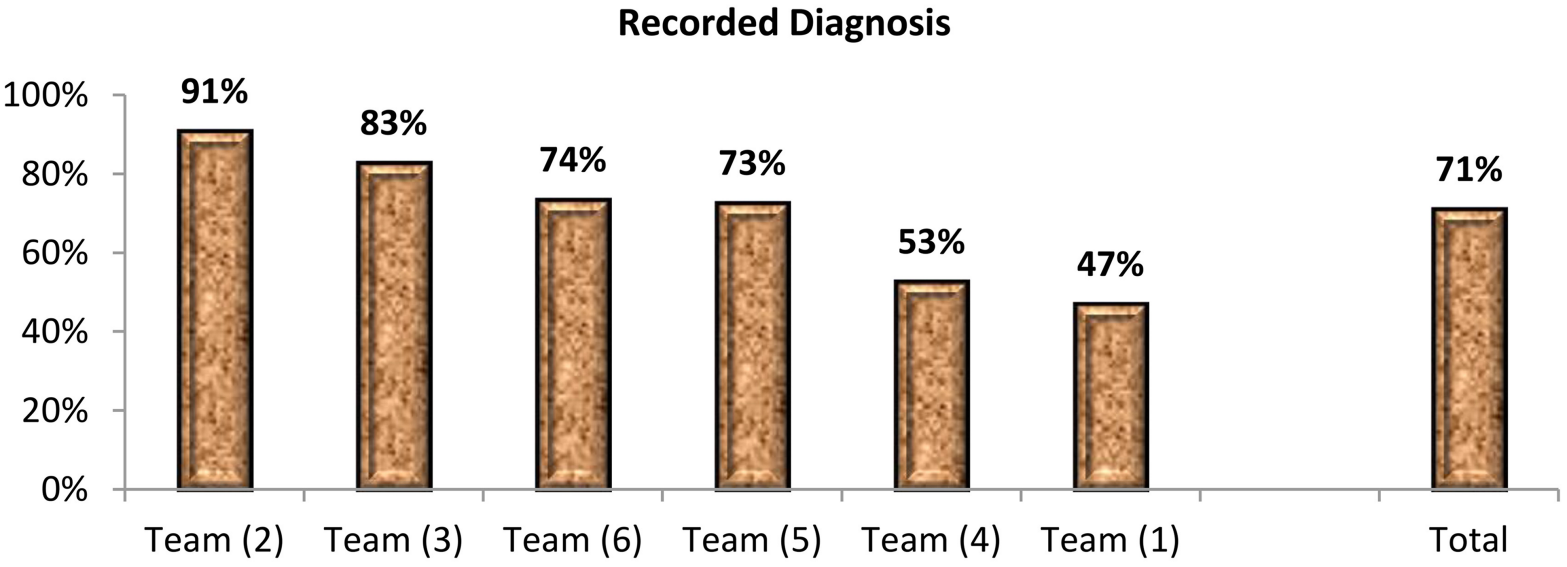
Rank order of the six teams of ophthalmologists according to the percentage of team members’ recording diagnosis in the patients’ files throughout 1 year (July 2017–June 2018) (total members = 222 physicians) at the Research Institute of Ophthalmology (RIO).

Percent of inpatient files with recorded patient diagnoses is an important indicator for the assessment of performance. Figure 3 shows that only 71% of patients' files fulfilled this indicator. However, the performance regarding such indicators varied across the six teams. Team 2 showed the highest percentage regarding recording diagnosis (91%) followed by Team 3 (83%) (Figure 3).

Supplementary Figure S3, Supplementary Data Sheet 1 reveals the percent ratio of inpatients to outpatients at the total and each team level. At the total level, the inpatient-to-outpatient ratio was 7% (i.e., out of each 100 outpatients, 7 cases were admitted). For Team 4, out of each 100 outpatients, 8 cases were admitted.

### 3.4 Surgical operations in RIO

**Figure 4:**
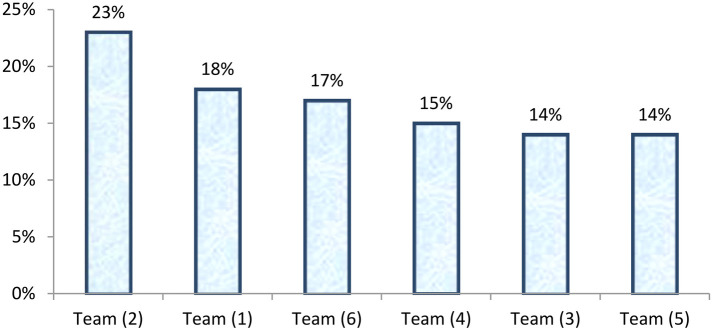
Rank order of the six teams of ophthalmologists according to the percent contribution in the total conducted surgical operations in 1 year (July 2017–June 2018) (total surgical operations = 9,174) at the Research Institute of Ophthalmology (RIO).

**Figure 5:**
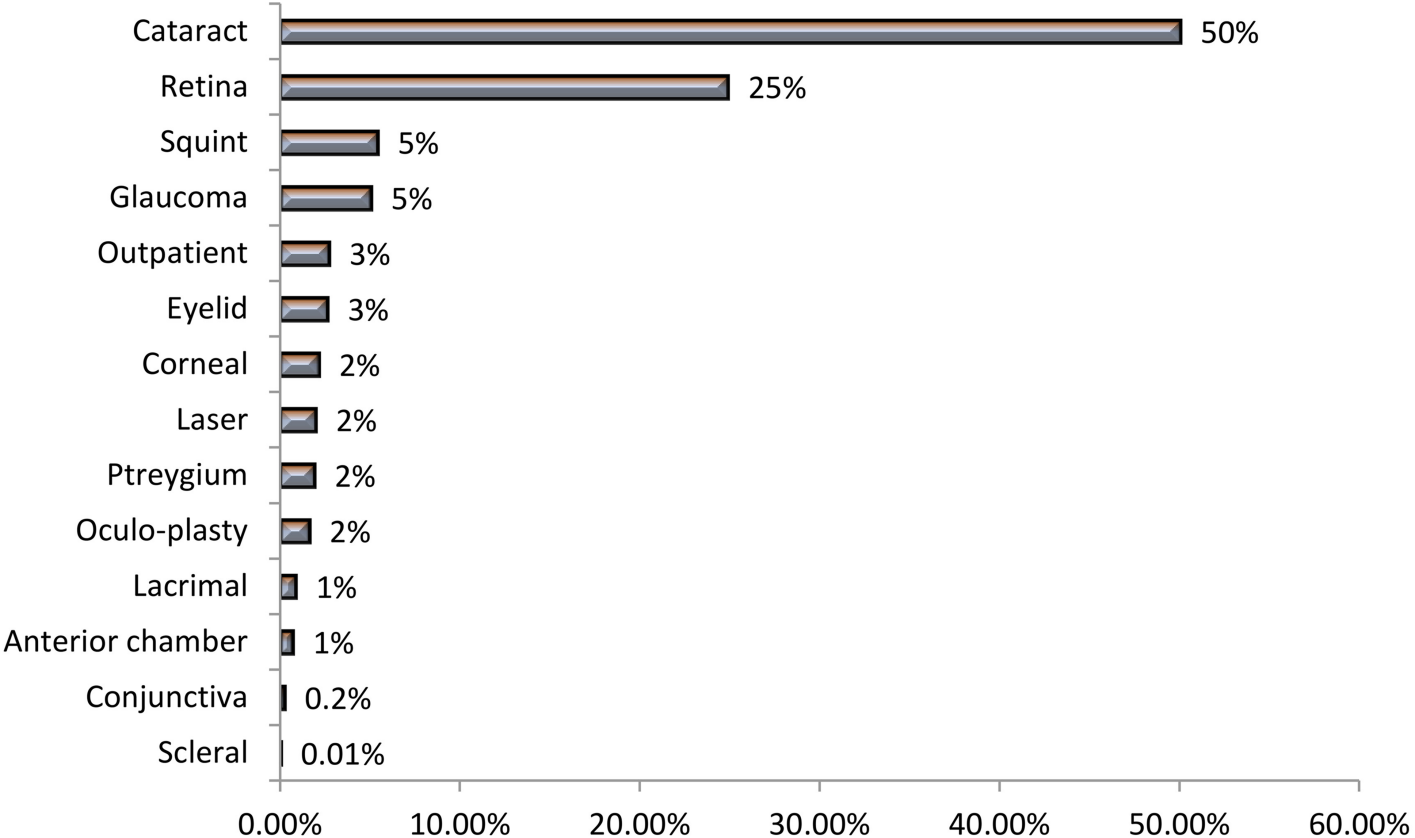
Percent distribution of the total surgical operations by anatomical category of operations from July 2017 to June 2018 at the Research Institute of Ophthalmology (RIO).

Throughout 1 year, from July 2017 to June 2018, and for 8,081 inpatients, a total of 9,174 surgical operations were done with an average of 1.1 surgical operations per case. Table 5 exemplifies a quarterly indicator of the percent contribution of the six teams of ophthalmologists to the total surgical operations. Team 2 contributed by 19%, 24%, 22%, and 25% of the total surgical operations, for the four quarters of the reference year, respectively. Teams 2 and 5 contributed by 23% and 14% of the total surgical operations, respectively. Out of the total 9,174 surgical operations, more than half (52%) were skilled surgeries, 27% were major surgeries, 13% were moderate surgeries, and 7% were minor surgeries. This profile varied across the six teams of ophthalmologists. For Team 3, 45% of its surgical operations were skilled surgeries, and 16% were minor surgeries. Team 2 contributed by 23% of the skilled surgeries and 21% of major surgeries. The table also illustrates the percent contribution of the six teams in total surgical operations by technical and anatomical category of operations at RIO throughout the same year. Teams 2, 3, and 6 had noticeable contributions in specific types of surgeries. A total of 14 types of surgical procedures were conducted at RIO during the same year. The indicator of the percent contribution of the six teams for the total 14 surgical operations was displayed in rank order of the six teams across types of surgical operations. The top teams of specific surgery specialties were Teams 2, 1, and 6. The percent contribution of the teams to the total surgical operations conducted in RIO from July 2017 to June 2018 is illustrated in Figure 4 showing Teams 2 and 5 contributing to 23% and 14% of the total surgical operations, respectively (Figure 4). Supplementary Figure S4, Supplementary Data Sheet 1 demarcated the profile of surgical operations in RIO throughout the same year. It illustrated that out of the total 9,174 surgical operations, more than half (52%) were skilled surgery, 27% were major surgeries, 13% were moderate surgeries, and 7% were minor surgeries. This profile varied across the six teams of ophthalmologists. For Team 3, 45% of its surgical operations were skilled surgery, and 16% were minor surgery. Figure 5 presents the ophthalmological operation in a technical and anatomical classification (Figure 5). Fifty percent of the surgical operations were cataracts, and 25% were retina surgeries. Rare surgical operations were conjunctiva surgeries at 0.2% and scleral surgeries at 0.01% (Table 5).

**Table 5:** Percent contribution of each team of ophthalmologists in total surgical operations throughout 1 year (July 2017–June 2018) and by skill and anatomical categories of surgical operations through the same year at the Research Institute of Ophthalmology (RIO).

Surgical operations 2018		Team 1	Team 2	Team 3	Team 4	Team 5	Team 6	Total
Quarters								
Q1		17%	19%	18%	18%	15%	15%	1,344
Q2		19%	24%	13%	15%	14%	15%	3,182
Q3		19%	22%	12%	14%	15%	17%	2,931
Q4		16%	25%	14%	12%	12%	20%	1,717
Skill categories of surgical operations								
Minor		17%	26%	30%	12%	8%	7%	673
Moderate		15%	23%	16%	18%	20%	7%	1,201
Major		21%	21%	12%	13%	12%	21%	2,492
Skilled		18%	23%	12%	15%	14%	18%	4,808
Anatomical Site specific	Code							
Cataract	C	20%	20%	11%	17%	15%	17%	4,590
Anterior chamber	AC	21%	30%	22%	6%	11%	10%	63
Scleral	S	100%	0%	0%	0%	0%	0%	1
Corneal	Corn	5%	36%	38%	7%	6%	7%	195
Glaucoma	G	24%	16%	48%	3%	7%	2%	462
Retina	R	15%	26%	9%	15%	19%	17%	2,282
Laser	L	8%	36%	9%	16%	16%	15%	179
Outpatient	OP	23%	30%	13%	17%	8%	10%	248
Pterygium	Pt	17%	16%	37%	12%	11%	8%	173
Conjunctiva	Conj	19%	29%	5%	24%	5%	19%	21
Squint	Sq	16%	21%	3%	14%	13%	33%	496
Eyelid	EL	20%	30%	23%	8%	1%	17%	240
Lacrimal	Lacr	26%	21%	26%	3%	0%	25%	77
Oculoplasty	Ocu	21%	27%	17%	13%	0%	22%	147
Total surgical operations		1,673 (18%)	2,080 (22%)	1,262 (14%)	1,347 (15%)	1,284 (14%)	1,528 (17%)	9,174 (100%)

AC, anterior chamber; C, cataract; Conj, conjunctiva; Corn, cornea; EL, eyelid; G, glaucoma; L, lacrimal; Ocu, oculoplasty; OP, outpatient; Pt, pterygium; R, retina; S, sclera; Sq, squint.

Supplementary Matrix S1, Supplementary Data Sheet 1 shows detailed information about the performance of each team in surgical operations classified as 14 technical and anatomical categories presented as percent distribution of surgical operations conducted by each team.

Supplementary Figure S5, Supplementary Data Sheet 1 demonstrates the performance of Team 1 regarding the percent distribution of surgical operations by technical and anatomical category. As depicted from the figure, the total number of surgical operations conducted by Team 1 was 1,673, from July 2017 to June 2018 with an average of 139 operations per month. Cataract and retinal surgeries accounted for 54% and 20%, respectively, of the total annual surgeries.

Supplementary Figure S6, Supplementary Data Sheet 1 demonstrates the performance of Team 2 concerning the percent distribution of surgical operations by technical and anatomical category. As depicted from the figure, the total number of surgical operations conducted by Team 2 was 2,080, from July 2017 to June 2018 with an average of 173 operations per month. Cataract and retina surgeries accounted for 45%.

Supplementary Figure S7, Supplementary Data Sheet 1 illustrates the performance of Team 3 vis-à-vis the percent distribution of surgical operations by technical and anatomical category. As depicted from the figure, the total number of surgical operations conducted by Team 3 was 1,262, from July 2017 to June 2018 with an average of 105 operations per month. Anterior chamber and retina surgeries accounted for 42% and 18%, respectively, of the total annual surgeries.

Supplementary Figure S8, Supplementary Data Sheet 1 displays the performance of Team 4 regarding the percent distribution of surgical operations by technical and anatomical category. As depicted from the figure, the total number of surgical operations conducted by Team 4 was 1,347 from July 2017 to June 2018 with an average of 112 operations per month. Conjunctiva surgeries and laser techniques accounted for 58% and 25%, respectively, of the total annual surgeries.

Supplementary Figure S9, Supplementary Data Sheet 1 reveals the performance of Team 5 regarding the percent distribution of surgical operations by technical and anatomical category. As depicted from the figure, the total number of surgical operations conducted by Team 5 was 1,284, from July 2017 to June 2018 with an average of 107 operations per month. Oculoplasty and glaucoma surgeries accounted for 52% and 33%, respectively, of the total annual surgeries.

The performance of Team 6 as regards the percent distribution of surgical operations by technical and anatomical category is illustrated in Supplementary Figure S10, Supplementary Data Sheet 1. As depicted from the figure, the total number of surgical operations conducted by Team 6 was 1,528, from July 2017 to June 2018 with an average of 127 operations per month. Pterygium and eyelid surgeries accounted for 51% and 26%, respectively, of the total annual surgeries.

### 3.5 Performance indices, decision matrices and total performance index

The key performance indices across the six teams of ophthalmologists are composed of:

F.1 Manpower resources index: The manpower (ophthalmologists) index is composed of three indicators calculated throughout 1 year in RIO: percentage of the total team members to total RIO's staff members, percentage of professors and assistant professors within the team to total professors and assistant professors, and percentage of trainees and fellowship scholars within the team to total trainees and fellowship scholars.

Supplementary Matrix S2, Supplementary Data Sheet 1 presents the rank ordering of each team of ophthalmologists according to three manpower resources indicators and index. Teams 2 and 3 were the top teams regarding the percent contribution of the staff members to the total RIO's staff members. Teams 3 and 6 were the uppermost regarding the proportion of professors and assistant professors to total professors and assistant professors. Teams 2 and 4 had a major role in including trainees and fellowship scholars among the team members. The matrix provided a conclusion that Teams 2 and 3 had the highest index regarding manpower resources.

F.2 Outpatient services index is composed of four indicators calculated throughout the same year in RIO: percent contribution of each team of ophthalmologists to total outpatient cases, percent contribution of each team to total outpatient cases whose age is less than 25 years, percent contribution of each team to total outpatient cases whose age is 55 years and more, and percent contribution of each team to total outpatient cases who attended for consultation services.

Supplementary Matrix S3, Supplementary Data Sheet S1 delineates that Teams 1 and 2 reported the highest achievement regarding the volume of the served outpatient cases and the highest contribution in consultation services.

F.3 Inpatient services index is composed of four indicators calculated throughout the same year in RIO: percent contribution of each team of ophthalmologists to total inpatient cases, percent contribution of each team of ophthalmologists to total inpatient cases defined as new cases (no previous admission to RIO), percentage of staff members in each team who recorded diagnosis in the inpatient files, and percent surgical operations conducted by each team to the total inpatients (coverage by surgical operations).

Supplementary Matrix S4, Supplementary Data Sheet S1 displays the performance of each team of ophthalmologists in inpatient services measured by the four indicators. Team 2 was keeping the top position in the 4 indicators that assess performance in the inpatient services.

F.4 Technical performance index is composed of three indicators calculated throughout the same year in RIO: percentage of surgical operations (14 categories) conducted by each team to the total surgical operations, percentage of surgical operations defined as “major surgery” conducted by each team to the total major surgical operations, and percentage of surgical operations defined as “skilled surgery” conducted by each team to the total skilled surgical operations.

Supplementary Matrix S5, Supplementary Data Sheet S1 illustrates that Team 2 was the top regarding the percent contribution in total surgeries, skilled, and major surgeries in RIO throughout 1 year.

F.5 General performance index is a composite index that includes four indices: manpower resources index, outpatient services index, inpatient services index, and technical/surgical operations index.

Matrix 1 articulates all indicators and indices of performance to present rank ordering of each team of ophthalmologists according to the total performance index. Team 2 was the top team in the total performance index, due to having the top position in the manpower resources index, inpatient services index, and surgical operations index.

## 4 Discussion

The current study is an operations research study that revealed the development of KPIs and indices and their application on data available in the RIO HMIS. It is concerned with the process and components of the management of healthcare services. The study is pivotal for any organization that could be a health or other service organization as it delineates steps for capitalizing on HMIS for timely decision-making to improve performance. Similar conducted studies depend on the development of KPIs related to hospitals (20). Some studies are based on the balanced scorecard model (16), and others are based on the analytic network process model (17), while in our study the manipulation of HMIS data for RIO was based on the type of operating health service. The data were reorganized and integrated for performance to be monitored and evaluated as team-focused. Most of the studies highlighted factors affecting the implementation of the HIS in hospitals depending on systematic review and qualitative data (18, 19), while our study investigated upgrading the MIS to improve performance. One of the studies used the census method to estimate the count of users of the HIS assuming that all clinical staff who had a bachelor’s degree and higher were using it (18). The current study relies on the actual number of physicians using the HIS through quantitative analysis of data derived from EMR. Hospital performance indicators usually included hospital bed utilization rates such as bed occupancy rate, bed turnover rate, and average length of hospital stay (26). However, those hospital performance indicators are not applicable in RIO, as the delivered services have the character of 1-day surgery and ultrashort hospital stay services. Some studies are hospital unit/department-oriented to link the HIS/MIS reports and well-defined departments (27), but this study developed horizontal indicators (relation of each team to the total of RIO services) and vertical indicators (performance at the team level).

To develop reports on providing ophthalmology services, the researcher was confronted with the situation that there is no standard or target to be achieved by each team. Therefore, the concept of using indicators across six teams was based on the assumption that both the resources and output are more likely to be equally distributed across the six teams with the percent contribution of each team to the total RIO output more likely to be 16.7%.

Previous studies mentioned HR indicators measuring HR from the perspective of their functional positions as the number of physicians (28), nurses, nurse assistants, operation room operators, and anesthesiologist assistants (29), clinical personnel (30), number of full-time equivalent interns/residents, administrative personnel, and non-clinical personnel (28) neglecting their distribution across teams/departments and their academic positions. The current study worked on the available data in the RIO HMIS regarding HR, which was related to the total number of physicians distributed across eight categories from the perspective of their academic positions [professors, professors emirate, assistant professors, researchers, researchers assistant, specialists (31), trainees and fellowship scholars (32)] and across six teams of ophthalmologists.

The human resource index (HRI) was previously mentioned by other studies as a staffing matrix (staffing number of positions filled, departmental turnover rates, and retention) and quality matrix (average tenure of employees, percentage of new hires retained for 90 days (22). Gu and Itoh (28) investigated the low awareness of hospital managers and staff by using employee development as an index considering the number of physicians and specialists as indicators. For each hospital bed in the Ministry of Health, medical universities, and hospitals in Iran, 0.85 nursing staff is measured as an index including nurse, nurse assistant, operation room operator, and anesthesiologist assistant as indicators (29).

In the current study, the six teams were ranked from 1 to 6. The best situation level for the team, i.e., had the highest percent contribution in any of the three indicators was provided a score of 6 and so on according to the rank ordering of teams for each indicator. The total score for each indicator and for each team is then divided by the maximum score (6 × number of indicators) to get the percent score for each team for a group of indicators. The percent scores for the teams were reallocated into three levels using centiles to have top teams, unfavorable situation teams, and intermediate-level teams. The index has no meaning in its value but it is used as a method to add different types of indicators together and to rank each team in the special management index, e.g., HR, outpatient, and others.

Indicators measuring performance in outpatient services mentioned in other studies include the average number of drugs per prescription and the proportion of drugs prescribed under inappropriate names (33). A study done on a tertiary care hospital in Saudi Arabia revealed the number of patients referred; number of patients on the waiting list for admission as patient access indicators; total number of outpatient clinic visits, new patients, follow-up patients, and new follow-up cases; and number of no show patients as outpatient utilization indicators and outpatient satisfaction rates (34).

The data available on RIO outpatient services were related to the total number of outpatient cases, age category, sex, and reason for seeking outpatient clinics, i.e., consultation or follow-up.

Indicators measuring performance in inpatient services are previously mentioned in other studies including average length of stay, bed occupancy rate, and bed turnover rate. The result of a study done on 15 hospitals indicated an increase in the average of all inpatient performance indicators such as average length of stay, bed occupancy rate, bed turnover rate, and inpatient rate after the implementation of a new health transformational plan (26). Managers of public hospitals were more aware of operational efficiency indicators than those of private hospitals (28). Inpatient mortality, readmission rate, pressure ulcer rate, discharge with personal satisfaction, clinical errors, and hospital infection rate were selected as indicators of the internal process perspective in the study of Rahimi et al. (15).

The data available on inpatient services in this study were related to the total number of inpatient cases, new and readmitted cases, ophthalmologists recording diagnoses in the inpatient files, and surgeries conducted in inpatient cases. The current study converted the data into indicators then selected four indicators defined as KPIs in inpatient services and then used them to estimate the inpatient performance index.

Indicators measuring performance in surgical operations mentioned in other studies include number of operation room (OR) cases booked, number of OR cases, performed number of OR cases, percentage of OR cancellations, and percentage of surgical operations to surgery beds as indicators investigating operation utilization and mortality rates measuring quality as investigated by Pourmohammadi (3). Surgical volume and percentage of surgeries classified by operative skills and specialties were compared in hospitals of Sierra Leone before and after an outbreak of the Ebola virus (35). Additionally, Khalifa and Khalid studied the OR utilization rate (34).

The data available on surgical operations were related to the total number of surgical operations, categories of surgical operations (minor, moderate, major, and skilled), and 14 types of operations by technical and anatomical classifications. The current study converted the data into indicators then selected three indicators defined as KPIs in surgical operations then used to estimate the surgical performance index.

There are many examples across the current study presented as matrices. For example, Supplementary Matrix S5 illustrates the indicators about the performance of RIO teams in surgical operations pointing to the unsatisfactory performance of Team 5 in surgical operations of RIO. Supplementary Matrix S2 is more likely to provide an answer to Team 5 situation as Team 5 ranked sixth among the six teams in the HRI.

From Table 1 with data about the percent distribution of ophthalmologists across six teams by professional category, Team 6 needed to increase the total number of ophthalmologists, especially specialists and trainees.

The decision must be made based on several criteria. For example, Matrix 1 indicates that Team 5 is the priority team for support in different aspects as it had ranked sixth among the six teams in the total performance index. Team 5 also ranked sixth in the HRI, inpatient index, and surgical operation index.

The decision matrix generated meaningful decisions based on evidence from well-defined indicators and indices. Decision in healthcare management is an information parameter that can be used by an organization's decision-makers to assess the current situation and observe trends over time (36).

The total performance index reflected the information that Team 2 was the topmost team in the total performance index, due to having the top position in the HRI, inpatient services index, and surgical operations index. The module that includes indicators and indices for six RIO teams is very flexible to include more indicators, indices, and teams or departments. For that reason, it could be used in any organization and over time with frequent updates. It was previously used by surveillance systems for infectious diseases for 27 governorates over 10 years (25). It was used also for 19 districts of the Giza governorate where human development indicators were used (24). In very large hospitals, the concept of having MIS for each specialty using KPIs and matrices could be used for monitoring and evaluation of each hospital specialty over time.

## 5 Limitations of the study

The currently available HMIS was designed by a special IT company. The concepts related to monitoring and evaluation of performance, epidemiological research, and operations research were not considered in the HMIS. Despite the HMIS system including overwhelming data especially in the revenue files, many data were missing. Additionally, articulation of data is not easy, namely, having surgical operations categories were 14 types in the operational files and 16 categories in revenue files with detailed information about each surgical operation regarding subtypes and revenue. For example, cataract operation had 18 subtypes, and each subtype had a specific value of revenue.

The missing data were related to patients regarding age, diagnosis, years of education, and residence, i.e., governorate and urban/rural.

The limitations identified in the current RIO HIS are related to the dissociation of data categories and irrelevance to the mission statement of RIO. Additionally, all recorded data were linked to each working physician who entered data on the provided services on certain days. Consequently, the number of performance reports issued for each physician and the number of reports depend on the number of physicians, which is influenced by the turnover of physicians.

## Data Availability

The raw data supporting the conclusions of this article will be made available by the authors, without undue reservation.
