# Supplementary material for: Promoting health information system in guiding decisions for improving performance: an intervention study at the Research Institute of Ophthalmology, Giza, Egypt
Source: Front Digit Health. 2024 Sep 18;6:1288776. doi: 10.3389/fdgth.2024.1288776 (PMC11444961; doi:10.3389/fdgth.2024.1288776)
Supplement: Supplementary file 1 [file Datasheet1.pdf]

Supplementary Material (Figures and Matrices)

1 Supplementary Figures

Supplementary Figure 1. Percent contribution of each of the six ophthalmologists' teams in the total staff members of Research Institute of Ophthalmology (n=222), July 2017- June 2018.

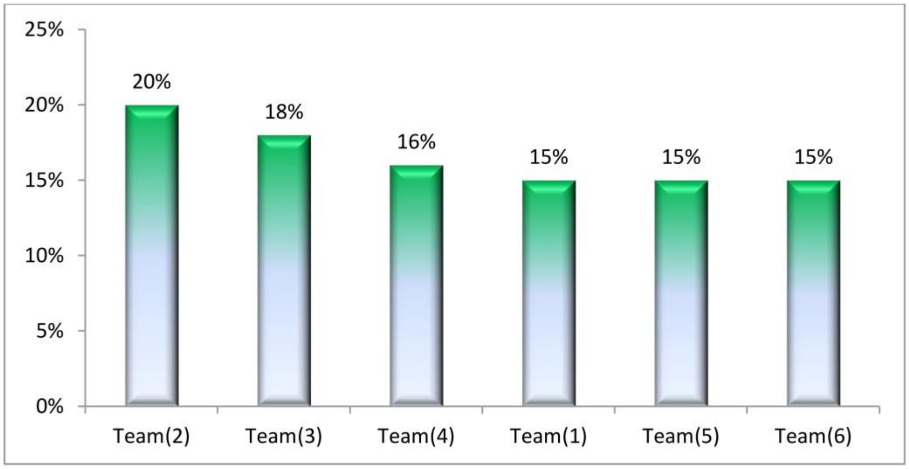

Supplementary Figure 2. Rank order of six ophthalmologists' teams according to proportion to the total staff members of professors and assistant professors (n=59) in Research Institute of Ophthalmology, July 2017- June 2018

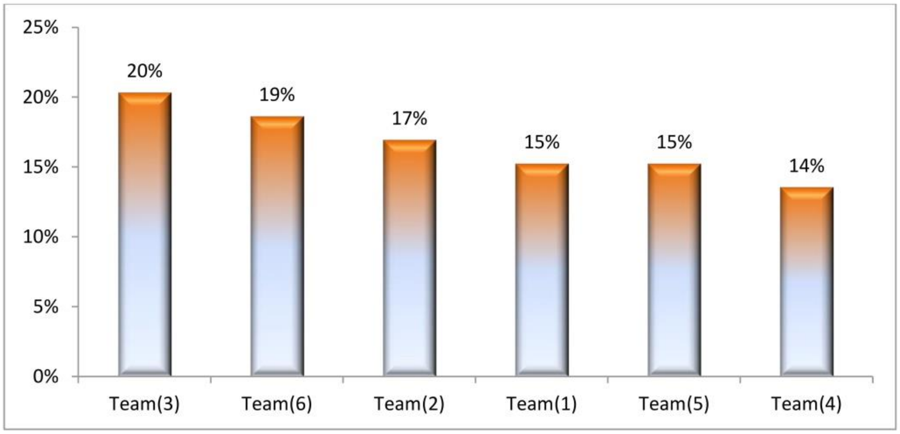

Supplementary Figure 3. Rank order of the six ophthalmologists' teams according to the percent of outpatient cases admitted to hospital throughout year 2017-2018, Research Institute of Ophthalmology

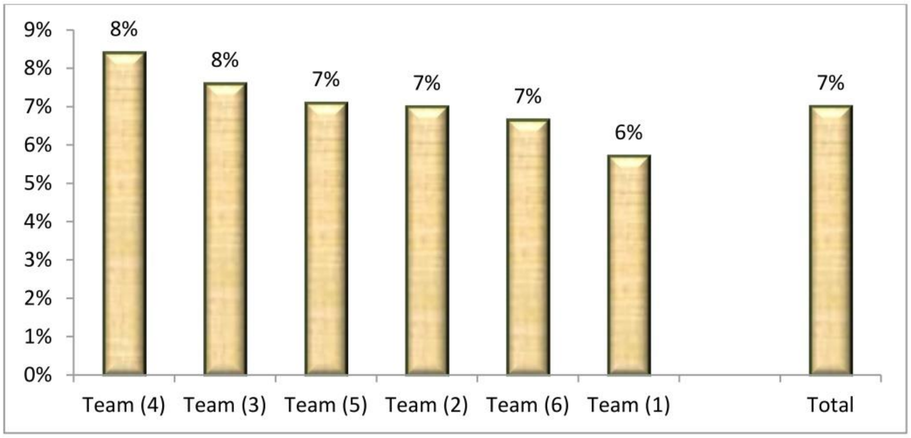

Supplementary Figure 4. Percent distribution of each of the six ophthalmologists' teams according to the skill categories of conducted surgical operations in one year July 2017-June 2018 (total surgical operations= 9174), Research Institute of Ophthalmology

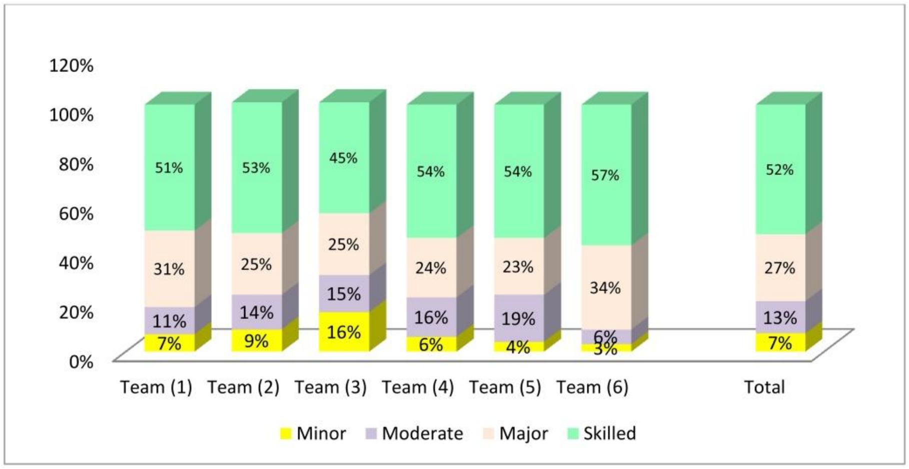

Supplementary Figure 5. Rank order of percent distribution of surgical operations conducted by Team (1) of ophthalmologists by Anatomical category of operations throughout one year July 2017-June 2018, Research Institute of Ophthalmology

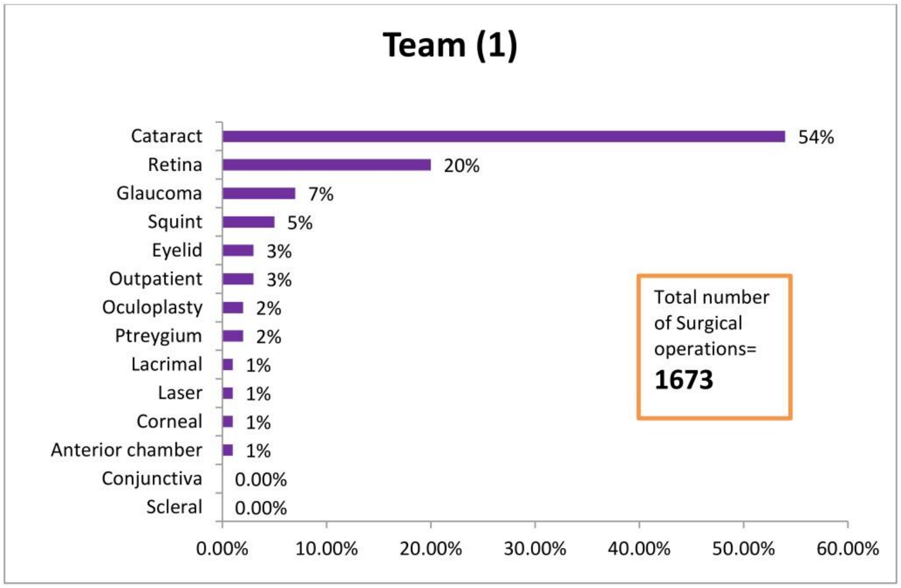

Supplementary Figure 6. Rank order of percent distribution of surgical operations conducted by Team (2) of ophthalmologists by Anatomical category of operations throughout one year July 2017-June 2018, Research Institute of Ophthalmology

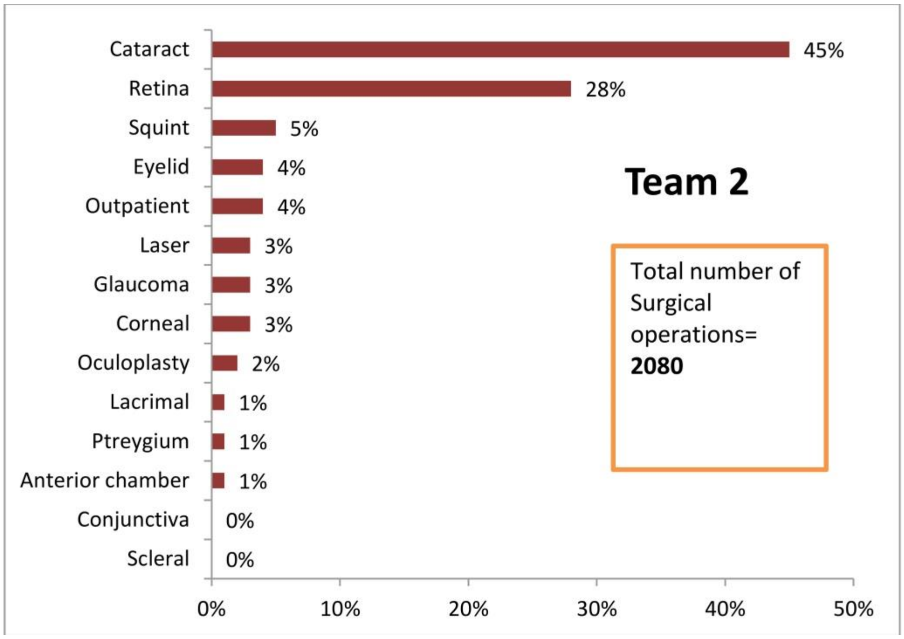

Supplementary Figure 7. Rank order of percent distribution of surgical operations conducted by Team (3) of ophthalmologists by Anatomical category of operations throughout one year July 2017-June 2018, Research Institute of Ophthalmology.

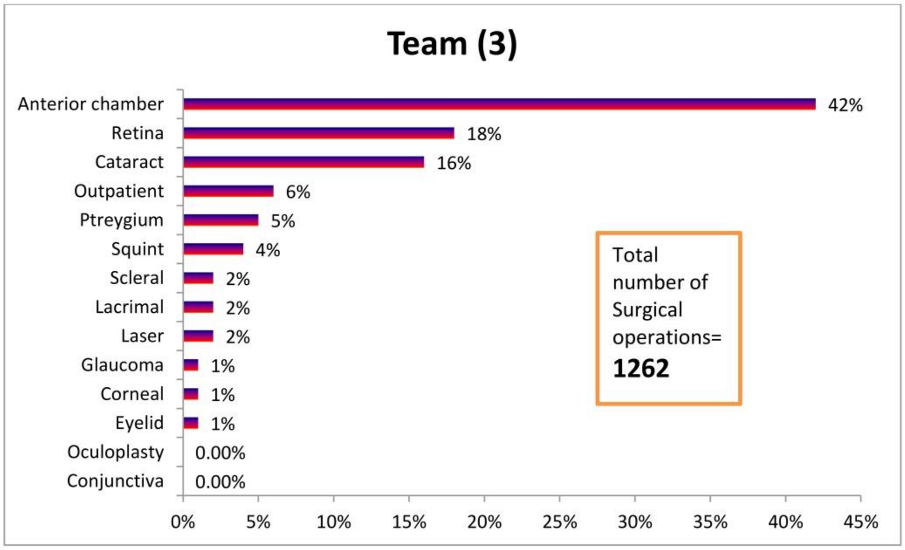

Supplementary Figure 8. Rank order of percent distribution of surgical operations conducted by Team (4) of ophthalmologists by Anatomical category of operations throughout one year July 2017-June 2018, Research Institute of Ophthalmology

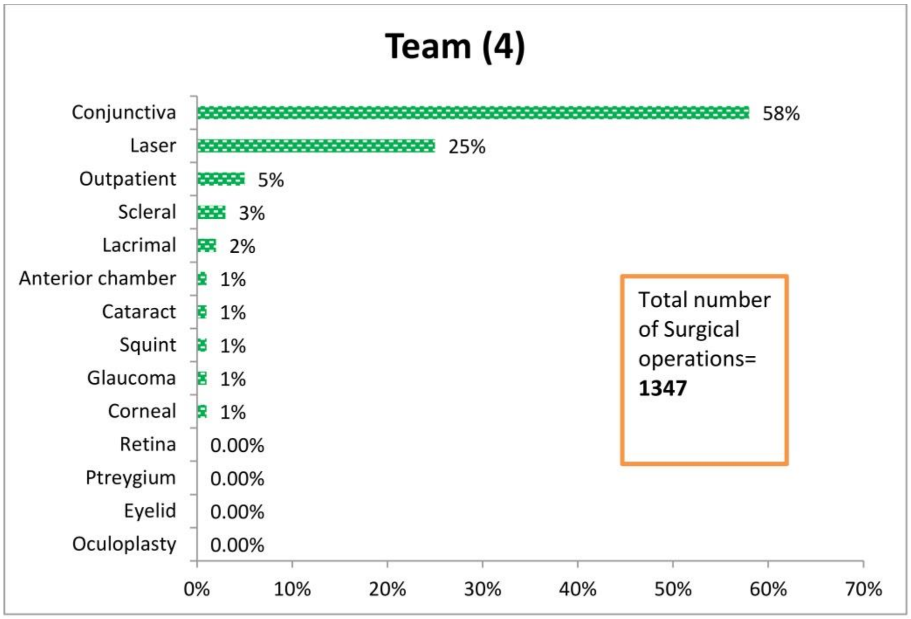

Supplementary Figure 9. Rank order of percent distribution of surgical operations conducted by Team (5) of ophthalmologists by Anatomical category of operations throughout one year July 2017-June 2018, Research Institute of Ophthalmology.

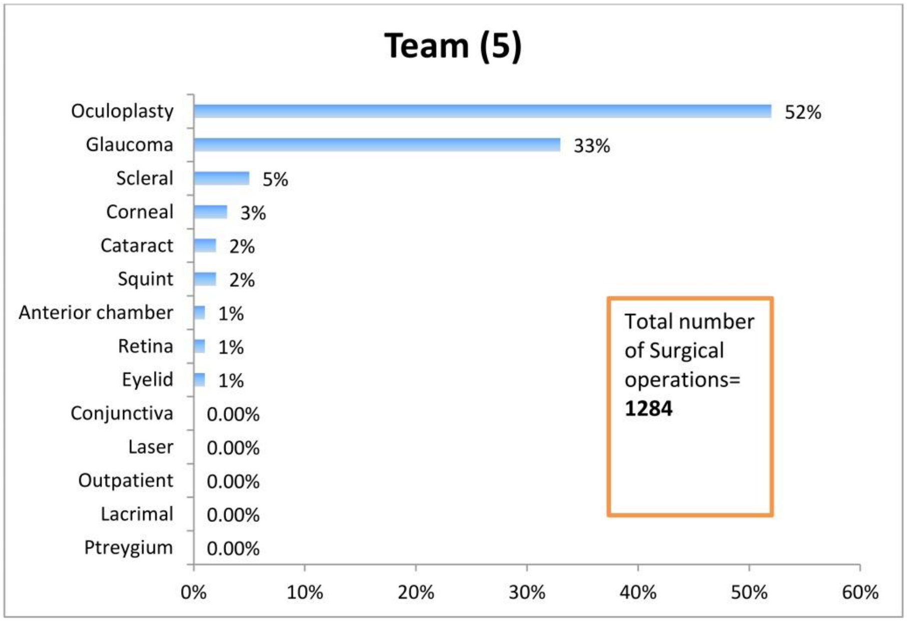

Supplementary Figure 10. Rank order of percent distribution of surgical operations conducted by Team (6) of ophthalmologists by Anatomical category of operations throughout one year July 2017-June 2018, Research Institute of Ophthalmology

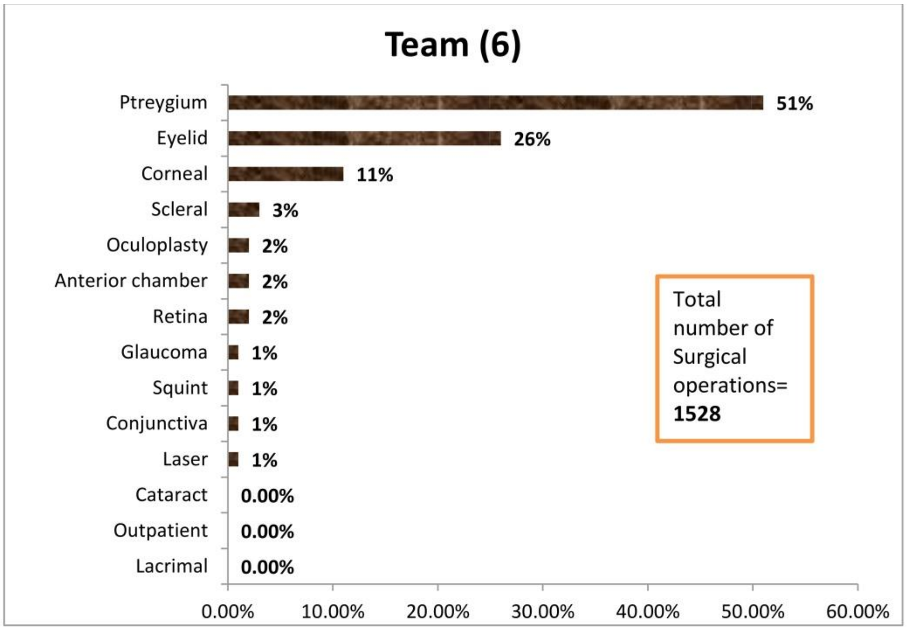

2 Supplementary Matrices

Supplementary Matrix 1. Rank positions of ophthalmologists' six teams for 14 surgical operations according to percent contribution to total surgical operations throughout one year July 2017-June 2018 Research Institute of Ophthalmology

Rank	Surgical Operations														Total
	C	AC	S	Corn	G	R	L	OP	Pt	Conj	Sq	EL	Lacr	Ocu	
1	T (1)	T (2)	T (1)	T (3)	T (3)	T (2)	T (2)	T (2)	T (3)	T (2)	T (6)	T (2)	T (1)	T (2)	T (2)
2	T (2)	T (3)	T (2)	T (2)	T (1)	T (5)	T (4)	T (1)	T (1)	T (4)	T (2)	T (3)	T (3)	T (6)	T (1)
3	T (4)	T (1)	T (3)	T (4)	T (2)	T (6)	T (5)	T (4)	T (2)	T (1)	T (1)	T (1)	T (6)	T (1)	T (6)
4	T (6)	T (5)	T (4)	T (6)	T (5)	T (1)	T (6)	T (3)	T (4)	T (6)	T (4)	T (6)	T (2)	T (3)	T (4)
5	T (5)	T (6)	T (5)	T (5)	T (4)	T (4)	T (3)	T (6)	T (5)	T (3)	T (5)	T (4)	T (4)	T (4)	T (3)
6	T (3)	T (4)	T (6)	T (1)	T (6)	T (3)	T (1)	T (5)	T (6)	T (5)	T (3)	T (5)	T (5)	T (5)	T (5)

Supplementary Matrix 2. Rank ordering of Ophthalmologists' teams according to three human resources' indicators, and index.

Total Human Resources indicator		Professors' indicator		Trainees and scholars indicator		Ophthalmologists Index	
Team 2	20%	Team 3	20%	Team(2)	22%	Team 2	89%
Team 3	18%	Team 6	19%	Team(4)	20%	Team 3	83%
Team 4	16%	Team 2	17%	Team(3)	17%	Team 4	56%
Team 1	15%	Team 1	15%	Team(6)	17%	Team 6	50%
Team 5	15%	Team 5	15%	Team(5)	15%	Team 1	39%
Team 6	15%	Team 4	14%	Team(1)	8%	Team 5	33%

Supplementary Matrix 3. Rank ordering of Ophthalmologists' teams according to 4 outpatient services indicators and index.

Percent contribution in Outpatient cases		Outpatients < 25 Years		Outpatients 55+ Years		Percent contribution in consultation services		Performance in outpatient services index	
Team 1	21%	**Team (2)**	**19%**	**Team (1)**	**20%**	Team 1	20%	Team 1	100%
Team 2	21%	**Team (6)**	**18%**	**Team (2)**	**20%**	Team 2	20%	Team 2	83%
Team 3	16%	**Team (1)**	**18%**	**Team (6)**	**16%**	Team 6	17%	Team 3	58%
Team 6	16%	**Team (5)**	**15%**	**Team (5)**	**15%**	Team 3	15%	Team 6	58%
Team 5	14%	**Team (4)**	**15%**	**Team (3)**	**15%**	Team 5	14%	Team 5	33%
Team 4	13%	**Team (3)**	**14%**	**Team (4)**	**14%**	Team 4	13%	Team 4	17%

Supplementary Matrix 4. Rank ordering of ophthalmologists' teams according to 4 indicators of performance in inpatient services and inpatient performance index

Total Inpatients		New Admissions		Recording Diagnosis		Surgeries/ inpatients		Inpatient Performance Index	
Team (2)	21%	Team (2)	20%	Team (2)	91%	Team (2)	26%	Team (2)	97%
Team (1)	17%	Team (1)	17%	Team (3)	83%	Team (1)	21%	Team (1)	72%
Team (3)	17%	Team (3)	17%	Team (6)	74%	Team (6)	19%	Team (6)	61%
Team (4)	16%	Team (6)	16%	Team (5)	73%	Team (4)	17%	Team (3)	47%
Team (6)	15%	Team (4)	15%	Team (4)	53%	Team (5)	16%	Team (4)	36%
Team (5)	14%	Team (5)	14%	Team (1)	47%	Team (3)	16%	Team (5)	36%

Supplementary Matrix 5. Rank ordering of ophthalmologists' teams according to 3 indicators of performance surgical operations services and Surgeries Performance Index

Total Surgical Operations and procedure		Major Surgical Operations		Skilled Surgical Operations		Surgeries Performance Index	
Team (2)	23%	Team (1)	21%	Team (2)	23%	Team (2)	94%
Team (1)	18%	Team (2)	21%	Team (1)	18%	Team (1)	89%
Team (6)	17%	Team (6)	21%	Team (6)	18%	Team (6)	67%
Team (4)	15%	Team (4)	13%	Team (4)	15%	Team (4)	50%
Team (3)	14%	Team (3)	12%	Team (5)	14%	Team (3)	28%
Team (5)	14%	Team (5)	12%	Team (3)	12%	Team (5)	22%
