# Supplementary material for: Promoting health information system in guiding decisions for improving performance: an intervention study at the Research Institute of Ophthalmology, Giza, Egypt
Source: Front Digit Health. 2024 Sep 18;6:1288776. doi: 10.3389/fdgth.2024.1288776 (PMC11444961; doi:10.3389/fdgth.2024.1288776)
Supplement: Supplementary file 2 [file Datasheet2.docx]

**Supplementary text.1 Description of the Parameters**

Despite having many indicators the researcher selected 14 specific indicators to be defined as key performance indicators. Those indicators were displayed in matrices for subsequent consolidation of indicators into 4 indices i.e. human resources, outpatient services, inpatient services, surgical operations and total performance index.

- The current study reformed the data into indicators then selected three indicators defined as key performance indicators in **human resources (HR)** and then used to estimate HR index.

The **first indicator** was defined as the percent contribution of each team in the total RIO-HR. Objective of the indicator is to measure the extent of equality in distribution of HR across the teams. Interpretation of the indicator that in case of having unequal distribution across the teams could reflect causes of variability in efficiency across the teams’ output services and revenue derived from team’s services. Limitations of the indicator: In case of increasing the total number of human resources in RIO, there will be ***no change*** in the value of the indicator for each team, unless redistribution of HR takes place in addition to increasing the total number of HR.

Relations with other indicators: This indicator has to consider other indicators as the percent distribution of total ophthalmologist’s team across the eight categories of the professional positions. Importance of the indicator in policy making: in case of increasing of human resources for the organization, proper distribution of human resources across teams /departments is mandatory. Example: The percent contribution of team 6 members to total RIO ophthalmologists was 15% , yet, 33% of total team 6 members (n=34) had the position of professors and assistant professors. The percent contribution of team 2 members to total RIO ophthalmologists was 20%, nonetheless, 22% of total team 2 members (n=44) had the position of professors and assistant professors.

The **second indicator** was defined as the percent contribution of professors and assistant professors in each of the six teams to total professors and assistant professors in RIO in the same time period.

Objective of the indicator is revealing strengths of each team regarding the volume of experts within each team. Interpretation of the indicator: the higher the value of this indicator, the higher the anticipation that such team would contribute to higher specific professional services. Limitations of the indicator: being a horizontal indicator, it does not reflect the structure of each team regarding the distribution of ophthalmologists by professional category. Relations with other indicators as in table (2) the ratio of professors to researchers and specialist in RIO was 1: 1.7. This ratio for the six teams was 1: 2.2 for team 1, 1: 2.2 for team 2 and 1: 1.6 for team 3 and 1: 1.9 for team 4 and 1: 1.7 for team 5 and 1: 1.5 for team 6. Importance of the indicator in policy making: in case of supporting human resources by new comers of professors, proper distribution of professors should be considered. Additionally, considering the ratio of the professors to researchers and specialist for each team should be deliberated. Team 2 with ratio of professors to specialists at 1: 2.2 had contributed to total RIO skilled surgical operations by 23%, which were the highest proportions compared with other teams.

The **third indicator** definition was percent sharing of trainees and fellowship scholars in each of the six teams to total trainees and fellowship scholars in RIO in the same time period. Objective of the indicator support the title and mission of RIO as it concerned with contribution in building the capacity of new generations in ophthalmology practice and clinical research. Relations with other indicators: as in table (2) the ratio of professors to trainees and fellowship scholars in RIO was 1: 1. This ratio for the six teams was 1: 0.6 for team 1, 1: 1.4 for team 2 and 1: 0.8 for team 3 and 1: 1.5 for team 4 and 1: 1 for team 5 and 1: 0.9 for team 6. Interpretation of the indicator: the higher the value of this indicator, the higher the role of the team in supporting the capacity building process for new generations. Importance of the indicator in policy making: in case of having new trainees and fellowship scholars, thoughtfulness should be directed to the distribution of professors across the teams to keep a reasonable ratio between professors and trainees.

- The current study reformed the data into indicators then selected four indicators defined as key performance indicators in **outpatient services** and then used to estimate outpatient performance index.

**First indicator**: Percent contribution of each team of ophthalmologists to total outpatient cases throughout one year. The objective of the indicator is to delineate the volume of outpatient services provided by each team. This indicator is related to other indicators as characteristics of the patients and reasons of seeking outpatient services. Also, this indicator could influence the proportion of outpatient cases admitted for surgical operations. Interpretation of the indicator: the high value of this indicator reflects success of the team in providing outpatient services to larger number of RIO outpatients. Limitations of the indicator: it is not influenced by increase in the total RIO outpatient services. The indicator reflects the teams’ competitions to contribute in total RIO services. Importance of the indicator in policy making: policy maker has to investigate the causes of low contribution of some teams to total outpatient services. Example (Figure 4) showed that the percent contribution of team 4 in outpatient services was 13%. The schedule for outpatient clinics could be revised, with adjusted in time management to provide opportunities for specific teams to increase the volume of outpatient services. However, monitoring of this indicator over time could provide different profile for each team.

**Second indicator**: Percent contribution of each team of ophthalmologists to total outpatient cases whose age less than 25 years -throughout one year. Objectives: identify the efforts of each team in providing ophthalmological services to young people. Interpretation of the indicator: the higher the value of this indicator, the higher the capacity of the team in providing ophthalmological services to young people. Due to lack of data related to age by diagnosis and major specialty of each team, it is difficult to set other related indicators. Importance of the indicator in policy making: The RIO could advocate for its role in providing ophthalmological services to young people who could be children or students or youth. This could promote for increasing resources especially for teams who had high value for this indicator. Example **(**Table 3): RIO outpatients in the age less than 25 years formed 29% of total outpatients. Team 5 provided outpatient services to the 32% of RIO outpatients who were less than 25 years old.

**Third indicator:** Percent contribution of each team of ophthalmologists to total outpatient cases whose age 55 years and more -throughout one year. Objectives: identify the efforts of each team in providing ophthalmological services to elderly. Interpretation of the indicator: the higher the value of this indicator, the higher the capacity of the team in providing ophthalmological services to geriatric group. Due to lack of data related to age by diagnosis and major specialty of each team, it is difficult to set other related indicators. Importance of the indicator in policy making: The RIO could advocate for its role in providing ophthalmological services to elderly people who could be disabled due to eye problems, having comorbidities as diabetes mellitus, hypertension, heart diseases and others. This group could be economically underprivileged due to being in the retirement age. This could promote for increasing resources especially for teams who had high value for this indicator. Example **(**Table 4): RIO outpatients in the age 55 years and more formed 34% of total outpatients. Team 5 provided outpatient services to the 36% of RIO elderly outpatients.

**Fourth indicator:**  Percent contribution of each team of ophthalmologists to total outpatient cases who attended for consultation services, throughout one year. The objective of the indicator is to demarcate the volume of outpatient consultation services provided by each team. This indicator is related to other indicators as characteristics of the patients and outpatient services for follow up. Interpretation of the indicator: the high value of this indicator reflects success of the team in providing outpatient consultation services to larger number of RIO outpatients. Limitations of the indicator is that it is not influenced by increase in the total RIO outpatient attending for consultation services. The indicator is linked with team competitions to contribute in total RIO outpatient consultation services. Importance of the indicator in policy making: policy maker has to consider the resources and auxiliary requirements (radiology, lab services etc.,) for teams with high value of this indicator Example ( table 5 and Matrix 3) showed that out of total RIO outpatients 90% sought consultation services and 10% attended for follow up services. The percent contribution of team 1 and 2 in outpatient consultation services were 20% each. However, team 1 outpatient services were 88% for consultation and 12% for follow up services. For team 2 outpatient services were 87% for consultation and 13% for follow up services

- **Inpatient services indicators:**

**First indicator:** Percent contribution of each team of ophthalmologists to total inpatient cases throughout one year. The objective of the indicator is to describe the volume of inpatient services provided by each team. This indicator is related to other indicators as characteristics of the patients, admission criteria, and human resources indicators. Also, this indicator could influence the proportion of inpatient cases for whom surgical operations were done. Interpretation of the indicator: the high value of this indicator reflects success of the team in providing inpatient services to larger number of RIO inpatients. Limitations of the indicator: it is not affected by increase in the total RIO inpatient services. The indicator is related to team competitions to contribute in total RIO inpatient services. Importance of the indicator in policy making: policy makers have to investigate the causes of low contribution of some teams to total inpatient services. Exampl**e** (table 7 and figure 6) showed that the percent contribution of team 5 and 6 in inpatient services was 14% 15% respectively. Teams 5 and 6 had as well lower level in the percent contribution of ophthalmologists to total RIO ophthalmologists (15% in figure 1).

**Second indicator:** Percent contribution of each team of ophthalmologists to total inpatient cases defined as new cases (no previous admission to Research Institute of Ophthalmology) throughout one year. The objective of the indicator is to demonstrate the capability of each team in attracting new cases with no previous admission to RIO. This indicator is related to other indicators as other types of admissions as readmission within 72 hours or after 72 hours. New admissions indicate that a package of services has to be provided to patients for diagnosis and management. Interpretation of the indicator: the high value of this indicator reflects success of the team in providing different services for diagnosis and treatment to larger number of RIO inpatients. Limitations of the indicator: it is not affected by increase in the total new RIO inpatient services. The indicator is allied to the teams’ competitions to contribute in total new RIO inpatient services. Importance of the indicator in policy making: policy maker has to investigate the causes of low contribution of some teams to total new inpatient services. However, in case of increase of total new in patients over time, the situation will reflect scaling up of RIO services due to good reputation. Example (table 6 and figure 5) showed that the percent contribution of team 5 in new inpatient services was 14% and team 6 by 16%. Teams 5 and 6 had as well lower level in the percent contribution of ophthalmologists to total RIO ophthalmologists (15% in figure 1).

**Third indicator** was percent of staff members in each team who recorded diagnosis in the inpatient files. This is a vertical indicator for each team. The objective is to identify commitments of ophthalmologists in each team to record the diagnosis in the inpatient files. This indicator is related **to** the percent of ophthalmologists in each team who do not record the diagnosis. According to the RIO available data which are physician- centered, it was difficult to develop a horizontal indicator. Additionally, the diagnosis could be recorded several times for the same case. This is because, the case could be diagnosed at the general outpatient clinic, and had another diagnosis at specialized clinic, then re-diagnosed after admission for inpatient services. Therefore, the recording diagnosis could be done for more than one time by different doctors. Consequently, the doctors who contribute in recording diagnosis could be detected from RIO-MIS data. Interpretation of this indicator in case of high value for specific team compared to other teams, delineates respectable performance of such team. Limitations of this indicator are related to sensitivity in case of relating non-recording to specific category of ophthalmologists. Importance of the indicator in policy making: Teams who had low proportion of ophthalmologists, who record the diagnosis in the patient files, should be informed about the importance of recording diagnosis. Example: Matrix 5 showed that 91% of team 2 members recorded the diagnosis in the patients’ files, versus team 1 where only 47% of its members report the diagnosis in the patients’ files.

**Fourth indicator:** Percent of surgical operations conducted by each team to the total inpatients (coverage by surgical operations) in Research Institute of Ophthalmology throughout one year.

Data for such indicator had been abstracted from two RIO-MIS folders, the surgical operation folder and the inpatient folder. The objective of the indicator is measuring the effectiveness of teams to provide surgical services to inpatients, in other words, the capability to cover inpatients with surgical operations (horizontal indicator). This indicator is related to other indicators as percent contribution of teams in inpatient services and surgical operations in RIO. In the current study, there were 9174 surgical operations to 8081 inpatients with inpatient: surgical operation ratio at 1:1.1. This ratio for the six teams was: 1: 1.2, 1: 1.2, 1: 0.9, 1: 1, 1: 1, and 1:1.2 respectively. Team 3 showed lowest coverage of inpatients with surgical operations as the ratio of inpatient to surgical operations was 1: 0.9 as a vertical indicator. At the horizontal indicator team 3 covered 16 % of all RIO inpatients and ranked number 6 in this indicator. Limitations of the indicators are related to the categories of surgical operations conducted by each team which are presented in other indicators. Importance of the indicator in policy making: Lower value of this indicator reflects improper time management and synchronization for admission-surgery accomplishment. Managers have to adjust timetables to help team 3 to increase admissions and activate the process of surgical operations

- **Surgical indicators:**

**First indicator:** Percent of surgical operations (14 categories) conducted by each team to the total surgical operations conducted in Research Institute of Ophthalmology throughout one year,

The objective of this indicator is to designate the volume of surgical services provided by each team. This indicator is related to other indicators as percent distribution of team to all inpatients, and human resources indicators. Interpretation of the indicator: the high value of this indicator reflects achievement of the team in conducting surgical operations at RIO level. Limitations of the indicator: it is not affected by increase in the total RIO surgical operations. The indicator is liked with teams’ efforts to contribute in total RIO surgical operations. Importance of the indicator in policy making: policy maker has to investigate the causes of low contribution of some teams to total surgical operations. Example (Figure 6, table 8 and figure 9) showed that the percent contribution of team 5 in inpatient services was 14%. Team 5 had as well lower level in the percent contribution in RIO total surgical operations (14%).

**Second indicator:** Percent of surgical operations defined as “major surgery” conducted by each team to the total major surgical operations conducted in Research Institute of Ophthalmology throughout one year. Objective is to identify the teams who provide outstanding surgical operations compared to other teams. This indicator is related to other vertical indicators related to the percent distribution of surgical operations conducted by the team according to category of surgical operation. Interpretation of the indicator that the higher the value of the indicator the great is the technical surgical performance of the team. Limitations: the value of the indicator was not influenced by increasing the total number of major surgeries conducted in RIO. Importance of the indicator in policy making: the teams who achieved high value for this indicator are considered very supportive to the role of RIO in providing tertiary level surgical care services. Policy makers are keeping interest to have an indicator that demarcates annual increase of major surgeries in RIO. Example: Out of the total RIO surgeries, 27% were major surgeries. Yet, out of the total team 6 surgeries , 34% were major surgeries. The contribution of team 6 to total RIO major surgeries was 21%.

**Third indicator:** Percent of surgical operations defined as “skilled surgery” conducted by each team to the total skilled surgical operations conducted in Research Institute of Ophthalmology throughout one year.

Objective is to identify the teams who provide spectacular surgical operations compared to other teams. This indicator is related to other vertical indicators related to the percent distribution of surgical operations conducted by the team according to category of surgical operation. Interpretation of the indicator that the higher the value of the indicator the great is the technical surgical performance of the team. Limitations: the value of the indicator is not influenced by increasing the total number of skilled surgeries conducted in RIO. Importance of the indicator in policy making: the teams who achieved high value for this indicator are considered very compassionate to the role of RIO in providing tertiary level surgical care services. Policy makers are keeping interest to have an indicator of annual increase of skilled surgeries in RIO. Example: Out of the total RIO surgeries, 52% were skilled surgeries. Nevertheless, out of the total team 6 surgeries 57% were skilled surgeries. The contribution of team 6 to total RIO skilled surgeries was 18%.
