# Supplementary material for: Promoting health information system in guiding decisions for improving performance: an intervention study at the Research Institute of Ophthalmology, Giza, Egypt
Source: Front Digit Health. 2024 Sep 18;6:1288776. doi: 10.3389/fdgth.2024.1288776 (PMC11444961; doi:10.3389/fdgth.2024.1288776)
Supplement: Supplementary file 3 [file Datasheet3.docx]

**Supplementary Material list**

**Supplementary Data**

1. **Supplementary Figures**

**Supplementary Figure 1.** Percent contribution of each of the six ophthalmologists’ teams in the total staff members of Research Institute of Ophthalmology (n=222), July 2017- June 2018.

**Supplementary Figure 2.** Rank order of six ophthalmologists’ teams according to proportion to the total staff members of professors and assistant professors (n=59) in Research Institute of Ophthalmology, July 2017- June 2018

**Supplementary Figure 3.** Rank order of the six ophthalmologists’ teams according to the percent of outpatient cases admitted to hospital throughout year 2017-2018, Research Institute of Ophthalmology

**Supplementary Figure 4.** Percent distribution of each of the six ophthalmologists’ teams according to the skill categories of conducted surgical operations in one year July 2017-June 2018 (total surgical operations= 9174), Research Institute of Ophthalmology

**Supplementary Figure 5.** Rank order of percent distribution of surgical operations conducted by Team (1) of ophthalmologists by Anatomical category of operations throughout one year July 2017-June 2018, Research Institute of Ophthalmology and 28% respectively for the total annual surgeries.

**Supplementary Figure 6.** Rank order of percent distribution of surgical operations conducted by Team (2) of ophthalmologists by Anatomical category of operations throughout one year July 2017-June 2018, Research Institute of Ophthalmology

**Supplementary Figure 7.** Rank order of percent distribution of surgical operations conducted by Team (3) of ophthalmologists by Anatomical category of operations throughout one year July 2017-June 2018, Research Institute of Ophthalmology.

**Supplementary Figure 8.** Rank order of percent distribution of surgical operations conducted by Team (4) of ophthalmologists by Anatomical category of operations throughout one year July 2017-June 2018, Research Institute of Ophthalmology

**Supplementary Figure 9.** Rank order of percent distribution of surgical operations conducted by Team (5) of ophthalmologists by Anatomical category of operations throughout one year July 2017-June 2018, Research Institute of Ophthalmology.

**Supplementary Figure 10.** Rank order of percent distribution of surgical operations conducted by Team (6) of ophthalmologists by Anatomical category of operations throughout one year July 2017-June 2018, Research Institute of Ophthalmology

1. **Supplementary Matrices**

**Supplementary Matrix 1.** Rank positions of ophthalmologists’ six teams for 14 surgical operations according to percent contribution to total surgical operations throughout one year July 2017-June 2018 Research Institute of Ophthalmology

**Supplementary Matrix 2.** Rank ordering of Ophthalmologists’ teams according to three human resources’ indicators, and index.

**Supplementary Matrix 3.** Rank ordering of Ophthalmologists’ teams according to 4 outpatient services indicators and index.

**Supplementary Matrix 4.** Rank ordering of ophthalmologists’ teams according to 4 indicators of performance in inpatient services and inpatient performance index

**Supplementary Matrix 5.** Rank ordering of ophthalmologists’ teams according to 3 indicators of performance surgical operations services and Surgeries Performance Index

1. **Supplementary Texts**

**Supplementary Text 1.** Description of the Parameters
